# A 60-year review on the changing epidemiology of measles in capital Beijing, China, 1951-2011

**DOI:** 10.1186/1471-2458-13-986

**Published:** 2013-10-21

**Authors:** Juan Li, Li Lu, Xinghuo Pang, Meiping Sun, Rui Ma, Donglei Liu, Jiang Wu

**Affiliations:** 1 Beijing Center for Disease Control and Prevention, 16 Hepingli Middle Road, Dongcheng District, Beijing 100013, China

**Keywords:** Measles, Epidemiology, Incidence, Vaccine, Coverage, Sero-epidemiology

## Abstract

**Background:**

China pledged to join the global effort to eliminate measles by 2012. To improve measles control strategy, the epidemic trend and population immunity of measles were investigated in 1951–2011 in Beijing.

**Methods:**

The changing trend of measles since 1951 was described based on measles surveillance data from Beijing Centre of Disease Control and Prevention (CDC). The measles vaccination coverage and antibody level were assessed by routinely reported measles vaccination data and twenty-one sero-epidemiological surveys.

**Results:**

The incidence of measles has decreased significantly from 593.5/100,000 in 1951 (peaked at 2721.0/100,000 in 1955), to 0.5/100,000 in 2011 due to increasing vaccination coverage of 95%-99%. Incidence rebounded from 6.6/100,000 to 24.5/100,000 since 2005 and decreased after measles vaccine (MV) supplementary immunization activities (SIAs) in 2010. Measles antibody positive rate was 85%-95% in most of years since 1981. High-risk districts were spotted in Chaoyang, Fengtai and Changping districts in recent 15 years. Age-specific incidence and proportion of measles varied over time. The most affected population were younger children of 1–4 years before 1978, older children of 5–14 years in 1978–1996, infant of <1 years and adults of ≥15 years in period of aim to measles elimination.

**Conclusion:**

Strategies at different stages had a prevailing effect on the epidemic dynamics of measles in recent 60 years in Beijing. It will be essential to validate reported vaccination coverage, improve vaccination coverage in adults and strengthen measles surveillance in the anticipated elimination campaign for measles.

## Background

Measles is a severe respiratory infectious disease caused by measles virus [1]. Measles has been statutorily notifiable since the earlier National Notifiable Diseases Reporting System (NNDRS) was established in 1950. Hospitals reported cases by posting a card to the county Center for Disease Control (CDC). Every month county CDC aggregated data that were then submitted through prefecture and provincial CDC to reach the national level [2]. In 1987 NNDRS was further improved, which reported basic epidemiologic data including age, sex, date of disease onset and residence by electronic document each month [3]. Since 2004 after severe acute respiratory syndrome (SARS), NNDRS was upgraded to direct reporting through network to improve timeliness, completeness of case reporting [4]. For each suspected case, the local CDC is required to carry out an epidemiological investigation, including obtaining specimens for laboratory confirmation since 2000.

Liquid measles vaccine (MV) was introduced in China in 1965. The 1978 establishment of the national Expanded Program on Immunization (EPI) resulted in a standard routine immunization schedule including MV administered as 1 dose to infants aged 8 months. In 1986, a 2-dose schedule using lyophilized MV at 8 months and 7 years of age was implemented [5]. The next step was a 1997 national plan of action for accelerated measles control that called for 90% MV coverage, measles surveillance and catch-up campaigns for provinces. In 2005, the World Health Organization’s (WHO) Regional Committee for the Western Pacific Region set a target date of 2012 for measles elimination in the region including China [6,7]. Since 2005, the age of administration of the second dose was lowered from 7 years to 1.5 years. The 2006–2012 national action plan to eliminate measles claimed comprehensive measures including 95% MV coverage of 2 doses, measles surveillance, regional and national supplementary immunization activities (SIAs), vaccination certificate of children at admission to child care setting and school [8,9]. China is a developing country with vast territory of 9.6 million square kilometers and large population of 1.37 billion, which has 23 provinces, 4 municipalities, 5 autonomous regions and 2 special administrative regions with different levels of economic and social development, predicting different challenges in reaching measles elimination. Hence, strategies of prevention and control measles were slightly different among different provinces.

**Figure 1:**
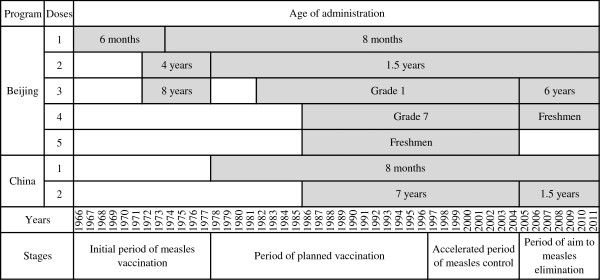
Measles vaccine immunization program of Beijing and China, 1966–2011.

Beijing is capital of China with a total area of 16,808 square kilometers. According to the 2010 national census, there were 19.6 million populations, of which 7 million migrants seek work in Beijing out of their province of origin. Referring to the historical periods of control of measles in China, combined with different strategies in Beijing, the strategies evolved from 1951 to 2011 in five stages (Figure 1):

Stage 1: Period prior to vaccination (1951–1965): Incidence of measles was in a natural infection state.

Stage 2: Initial period of measles immunization (1966–1977): MV was administered as 1 dose to infants aged 6 months. The second and third doses were administered to children of 4 and 8 years old in 1972. The age of administration of the first dose was increased to 8 months in 1974. Vaccine cold chain was not established. No refrigerant equipment was available and vaccine could not be stored at proper temperatures. In order to ensure vaccine immunogenicity, children with a certain age were gathered to vaccination twice each year in winter and spring.

Stage 3: Period of planned immunization (1978–1996): The 1978 establishment of the Beijing EPI resulted in a standard routine immunization schedule as a 2-dose schedule at 8 months and 1.5 years of age. Since 1982, Jing_55_ MV strains were replaced by Hu_191_ strains to improve vaccine immunogenicity. MV immunization population was expanded to students in Grade 1 and 7 in school, freshman in college. In 1987, the change of MV dosage form from liquid to lyophilized improved vaccine stability. With the gradually improvement of vaccine cold chain, children could be vaccinated in the whole year with the establishment of medical institutions designated inoculation. The subsequent expansion of cold chain infrastructure allowed routine immunization to cover the entire country.

Stage 4: Accelerated period of measles control (1997–2004): Since 2000, SIAs among migrant children under 7 years old was implemented every year. Measles vaccination dose was increased from 0.2 ml to 0.5 ml to enhance vaccine immunogenicity in 2004.

Stage 5: Period of aim to measles elimination (2005–2011): Since 2005, the vaccination schedule was recommended 3-doses vaccine schedule at 8 months, 1.5 and 6 years of age with univalent MV in all districts. Measles, rubella and mumps combined attenuated live vaccine (MMR) and measles and rubella combined attenuated live vaccine (MR) were introduced into the immunization program in 2006 and 2009, respectively. Measles vaccination campaign targeted migrant workers aged 15–40 years was implemented every year. The synchronized nationwide SIAs targeting children aged 8 months to 14 years was conducted in 2010.

Global vaccination programs have led to a worldwide decrease in the occurrence of measles [10,11], with some areas documenting an interruption of transmission of the endemic virus [12,13]. This study aimed to review the changing epidemiology of measles, measles vaccination coverage in relationship to the trend of measles incidence, herd immunity of the population against measles through periodic sero-epidemiological surveys from 1951 to 2011 in Beijing.

## Methods

### Case definition and source of data

Data of measles cases and deaths in 1951–2011 were obtained from Beijing CDC. Suspected measles cases was diagnosed clinically as a person of any age with fever, rash and one of the following: cough, coryza or conjunctivitis, classified as either laboratory-confirmed, epidemiologically linked, clinically confirmed or discarded, by guidelines of World Health Organization [14,15]. With the improvement of surveillance and moving towards measles elimination, the demands of measles case classification have changed during different phases. Before period of planned vaccination in 1978, almost all measles were clinical diagnosed case due to lack of routine and convenient laboratory test method. Since 1980, laboratory test method of measles antibody was used in case diagnosis. The national serological surveillance laboratory network was established in 2001 to provide serological confirmation of measles infection and to support measles surveillance. All cases included in this analysis were confirmed cases including laboratory or clinical confirmed and epidemiological related cases. Annual incidence and morbidity (per 100,000 populations) were calculated based on Beijing census data reported by the statistical yearbook of Beijing.

### Vaccination coverage

Administrative data based on report from health centre staff were the main sources of immunization coverage. The doses of vaccines administered in a given period (usually 1 month) were reported to prefecture CDC, in which data were collected and reported to Beijing CDC. In Beijing CDC, data were aggregated to estimate the average coverage rate using the numerator (the total number of actually immunized children) divided by the denominator (the total number of children who should be immunized by immunization schedule). For example, the 1978 establishment of the Beijing Expanded Program Immunization (EPI) recommended a standard routine immunization schedule as a 2-dose MV at age of 8 months and 1.5 years. The target population of coverage rate of first and second doses was children aged more than 9 and 19 months old, respectively, who lived in Beijing for more than 2 months. Vaccine immunization data from 1971 to 2011 were obtained from Beijing CDC.

### Sero-epidemiological surveys

In order to assess the level of immunity of the population against measles, sero-epidemiological surveys were conducted in 1973–1977, 1979-1982, 1986, 1989, 1990, 1995-2000, 2002, 2003, and 2007 in selected districts of Beijing. The sampling procedure was based on a three-stage protocol. Firstly, at least 3 prefectures were selected as random or specific survey sites from downtown, suburban and rural areas. Secondly, the villages were sampled by probability-proportional-to-size sampling (PPS). Thirdly, a number of objects per birth cohort were selected by convenience sampling method in the local population. Blood samples were collected from healthy persons with consent. Information integrated by the time of blood collection including basic demographic data, history of previous measles infection and MV vaccination. The blood samples were analyzed for measles IgG antibodies using measles enzyme immunoassay. Measles sero-epidemiology data from 1973 to 2007 were obtained from Beijing CDC. Sero-epidemiology surveys were performed with the approval of Beijing CDC ethics committee.

### Statistical analyses

We analyzed the data of confirmed measles cases reported in Beijing in 1951–2011 to identify epidemiological changing features, spatial geographic and age distribution, vaccination coverage and measles antibody positive rate. We used Microsoft Excel 2000, SPSS 17.0 and Mapinfo 8.5 for data analysis.

## Results

### Incidence and mortality of measles in Beijing

**Figure 2:**
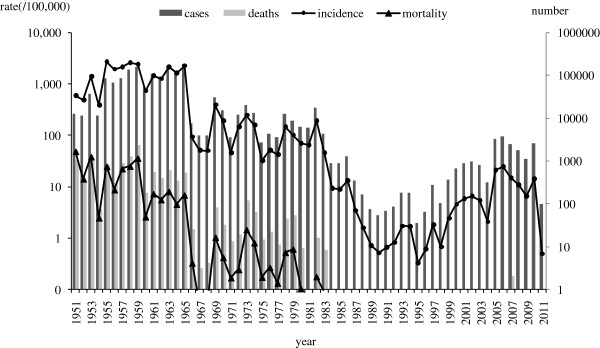
Measles incidence and morbidity in Beijing, 1951–2011.

Overall, 1,507,956 reported cases and 11,130 deaths of measles were recorded in Beijing over the 60-year period. Figure 2 showed the temporal pattern of the incidence and mortality of measles in Beijing in 1951–2011. The overall declining trend was clear, with the annual average incidence decreasing from 593.5/100,000 in 1951 to 0.5/100,000 in 2011 and mortality declining from 48.4/100,000 in 1951 to 0 in 2011. From 1951 to 1965, measles incidence fluctuation ranged from 390.7/100,000 to 2721.0/100,000. A frequent peak of incidence was observed every 2–3 years. Measles mortality was high, ranging from 2.5/100,000 to 48.4/100,000. In 1966–1977, incidence ranged from 32.2/100,000 to 397.1/100.000, which declined 92.7% compared with incidence before vaccination. There was a 3–4 year periodic recurrent pattern. Mortality declined significantly to 0.1-1.5/100,000. In 1978–1996, there were two phases in variation of incidence. Before 1985, average incidence was 78.7/100,000. Since 1986, the incidence of measles has remained at a relatively low level, less than 2.0/100,000 in most of years. No measles deaths were reported from 1986 to 1996. In 1997–2004, incidence was between 0.7/100,000 and 6.6/100,000. In 2005–2011, incidence rebounded from 6.6/100,000 to 24.5/100,000. After MV SIAs in 2010, incidence decreased to 0.5/100,000 in 2011. 4 deaths were reported in 1997–2011.

### Age distribution of measles

**Figure 3:**
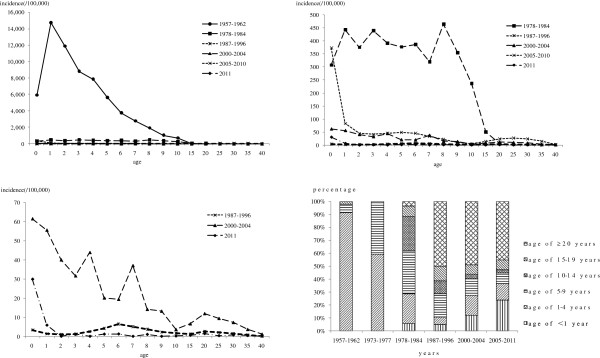
**Age-specific incidence and proportion of measles in Beijing, 1957–2011.** The measles cases by age groups in 1963–1972, 1985–1986 and 1997–1999 and the age-specific incidence in 1963–1977, 1985–1986 and 1997–1999 were not recorded in detail.

Age-specific incidence of measles varied over time. In 1957–1962 prior to vaccination, high measles incidence, 685.5/100,000 to 14,743.2/100,000, was found in children aged ≤14 years, especially among children aged 1–2 years. Incidence decreased gradually by age. After the introduction of initial planned vaccination in 1978–1984, incidence decreased dramatically from 237.0/100,000 to 465.3/100,000 in children aged ≤14 years. In 1987–1996, incidence declined continually in all age groups, ranging from 0.1/100,000 to 6.4/100,000. Since this period, age-specific incidence showed a change, three peaks of higher incidence were found in infants of <1 years, children of 5–7 years and adults of 20–30 years. In 2000–2004, incidence increased in all age groups, ranging from 3.6/100,000 to 61.5/100,000 for children aged ≤14 years and 1.1/100,000 to 11.9/100,000 for adults aged ≥15 years, with two crest in infants of <1 years and adults of 20–30 years. In 2005–2010, age-specific incidence increased to 5.1/100,000-82.9/100,.000 for children aged ≤14 years and 2.3/100,000-28.0/100,000 for adults aged ≥15 years, with highest 373.0/100,000 in infants aged <1 years. After MV SIA in 2010, incidence declined greatly to 0–1.2/100,000 for ≥2 years age group, while incidence was 29.9/100,000 for <1 years age group and 5.8/100,000 for 1–2 years age group (Figure 3).

Age-proportion distribution of measles cases varied over time. From 1957 to 1962, the most affected age groups were children of 1–4 years old, with proportion of 91.0%. The proportion of 5–9 years was 7.0%. From 1973 to 1977, the proportion of measles cases decreased to 59.1% in children of 1–4 years, but increased to 40.7% in children of 5–9 years. From 1978 to 1984, the proportion of 1–4 years and 5–9 years age groups were continually decreased to 23.2% and 33.0%, respectively. The affected age groups expand to 10–14 years age group with 26.4% and ≥15 years with 12.0%. In 1987–1996, the proportion of 1–14 years age group decreased to 5.6%-18.0%, while the proportion of adults of ≥15 years increased to 61.4%. In 2000–2004, proportion for infants of <1 years and children of 1–4 years increased to 11.8% and 15.6%, gradually. Adults of ≥15 years accounted for 56.5%. In 2005–2011, proportion of infants aged <1 years continually increased to 23.5%. Adults aged ≥15 years accounted for 52.9% (Figure 3).

### Spatial distribution of measles incidence

**Figure 4:**
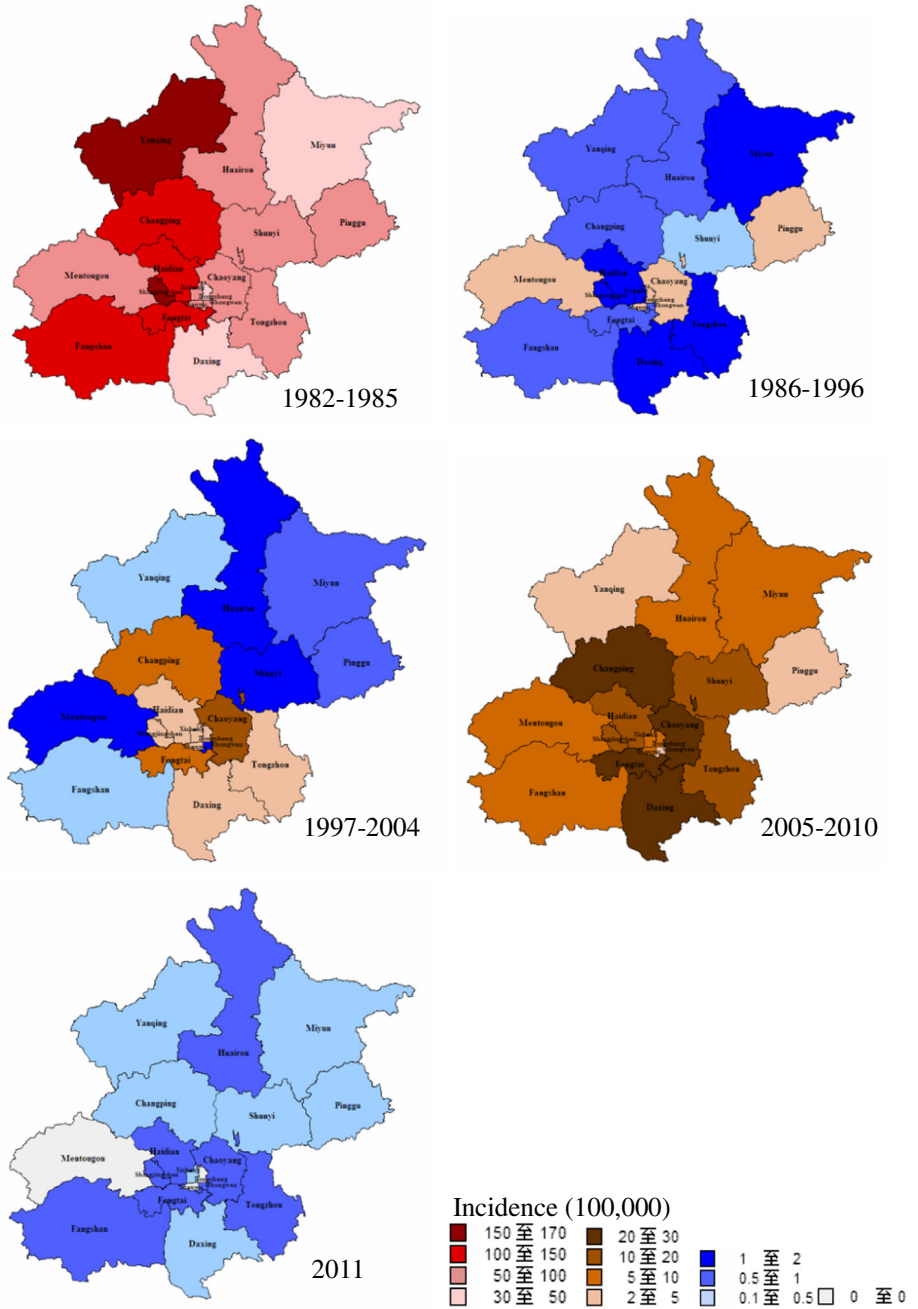
Spatiotemporal distribution of measles incidence in different stages, 1982–2011.

Measles was observed in all districts in Beijing, while the spatial distribution varied from 1982 to 2011 (Figure 4). In 1982–1985, measles broke out in all districts and the incidence range from 38.3/100,000 to 160.2/100,000. In 1986–1996, measles incidence decreased dramatically in all districts, ranging from 0.2/100,000 to 3.6/100,000. In 1997–2004, incidence of all districts remained lower level at 0.1/100,000 to 10.5/100,000. In 2005–2010, incidence in all districts increased slightly from 2.1/100,000 to 27.3/100,000. The main high risk districts were Chaoyang, Fengtai and Changping. After MV SIAs in 2010, incidence in all districts declined dramatically to lowest level, which was 0 in three districts.

### Seasonality of measles

**Figure 5:**
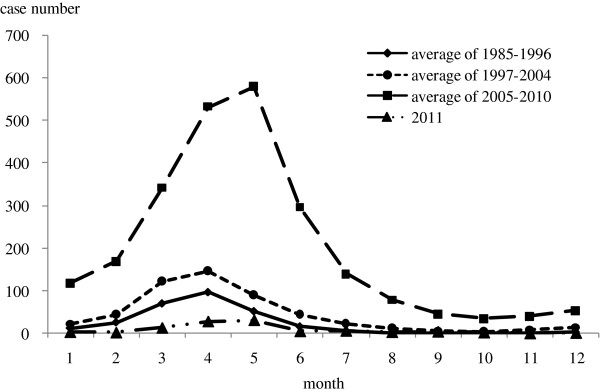
**Seasonality of measles in 1985–2011.** The reported cases number by month before 1985 was not recorded in the historical documents.

Peaks in the reported measles mainly occurred from March to May in 1985–2010, while it was no clear seasonal trend in 2011 when incidence declined to the lowest level (Figure 5).

### Vaccination coverage of measles vaccine

**Figure 6:**
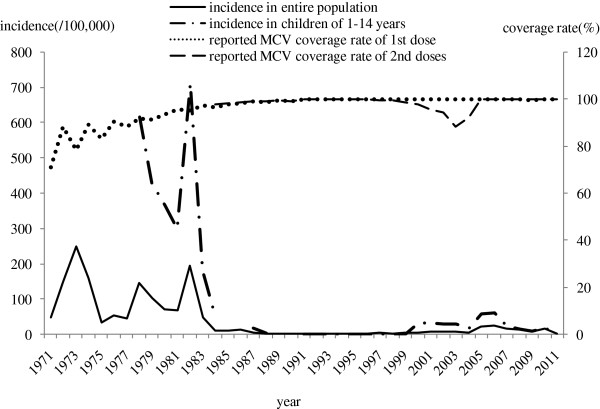
Measles Incidence and immunization coverage rate in Beijing, 1971–2011.

From 1971 to 1977, the MV coverage rate of first dose increased from 71.2% to 88.6%, while measles incidence remained high level during this period. During period of planned vaccination in 1978–1996, the MV coverage rate of first and second doses increased gradually from 91.2% to 99.9%, and measles incidence in entire population decreased dramatically from 146.0/100,000 to 0.3/100,000 and incidence in children of 1–14 years also declined greatly from 616.7/100,00, to 0.1/100,000. During accelerated period of measles control from 1997–2004, the MV coverage rate of second dose appeared a decline around 88.4% -94.5% in 2002–2004, which was probably related to rebound of measles incidence of whole population and children of 1–14 years in 2005 (Figure 6).

### Measles sero-epidemiology

**Figure 7:**
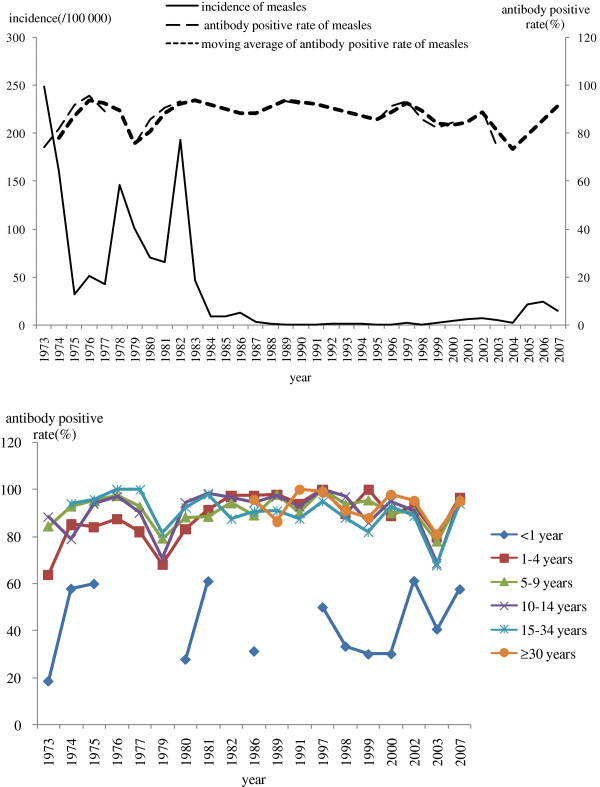
Measles Incidence and antibody positive rate in Beijing, 1973–2007.

From 1973 to 1977, antibody positive rate of measles increased from 73.3% to 95.8%, while incidence decreased from 248.7/100,000 to 42.2/100,000. Antibody positive rate of children 1–4 years was relative low, 63.8%-87.5%, when the first dose of MV was administrated at 6 months. Antibody positive rate of children 5–9 years was increased to 84.3%-97.4%, when the second and third dose of MV was administrated at 4 and 8 years old. From 1978 to 1996, antibody positive rate increased from the lowest 75.7% to higher 93.6%, while incidence declined from highest 146.0/100,000 in 1978 to lowest 0.3/100,000 in 1995. Age-specific antibody positive rate of 1–4 years was increased greatly from 68.0% to 97.9% while the first and second dose of MV was administrated at 8 months and 1.5 years old. Antibody positive rate increased from 70.6% to 98.0% for 5–14 years old , while students in Grade 1 and 7 was administrated MV from 1982. From 1997 to 2004, measles antibody positive rate fall to 93.4% and 73.4%, while incidence appeared slight rebound. During this period, age-specific antibody positive rate of 1–14 years was between 85.7% and 100%, except 68.6%-80.0% in 2003. Range of antibody positive rate in infants <1 years was 30.0%-61.2% (Figure 7).

## Discussion

Based on the trend analysis data from previous 60 years in this study, the measures of prevention and control measles at different stages had a tremendous effect on the epidemic dynamics of measles. Consistent with observations from other countries, epidemic cycles of measles occurred every 2–3 years in Beijing before measles vaccination, when measles virus was in a natural infectious state. Virtually everyone experienced measles illness during the childhood [16]. 91% of individuals were infected by the age of 1–4 years. After implementation of measles vaccination, especially program immunization with two doses at age of 8 months and 1.5 years in 1978, measles incidence drop dramatically [17,18], but resurgences occurred in 1982 and again in 1986. These resurgences may result from low effectiveness of liquid MV from Jing_55_ strains [19] and an accumulation of susceptible to a critical threshold level. This highlighted that situation of lower immunogenicity and administration of two doses was inadequate for the interruption of measles transmission. Jing_55_ MV strains were replaced by Hu_191_ strains, which had good immunogenicity and long-term persistence [20] and the third dose was introduced since 1982. Furthermore in 1987, liquid MV was replaced by lyophilized vaccine to improve vaccine stability and immunogenicity. Increasing vaccination coverage led to declining incidence of measles to historical lowest level in 1995 and longer inter-epidemic periods of 3–4 years. The incidence of measles appeared rise from 2005 due to enhancement of measles surveillance susceptibility through directly reporting suspected measles cases by network [21]. The 2010 national MV SIAs targeting children aged 8 months to 14 years was successful in largely interrupting measles virus transmission, resulting in almost historical lowest level of measles incidence in 2011.

With the increasing vaccination coverage in Beijing, the age distribution of cases shifted from younger children of 1–4 years to older children of 5–14 years and adults of 15–30 years; The upward shift in the age of measles cases to older age groups with increasing MV coverage was well documented in other settings [22-25].Toward the end of endemic measles virus transmission in the Americas, outbreaks in some regions included cases among older children and young adults [26,27]. In Beijing, young adults, especially migrant workers from other province living in the poor and crowd environment, clearly played a large role in the initial spread of measles epidemic. China could be divided into 3 groups: eastern, central and western provinces or regions, according to economic and social development. Migrants leaved their hometown in western and central provinces including to Qinghai, Gansu, Yunnan, Sichuan, Hunan, Hubei, Henan, and Anhui, et al. and swarmed to the eastern and more developed provinces including Beijing, Shanghai, Guangdong, Zhejiang, et al. in recent two decades. The national measles surveillance indicated that the average reported measles incidence decreased more in the Western (from 131.2/100,000 to 105.8/100,000) than in the Central provinces (from 57.6/100,000 to 55.5/100,000) from 1990–1999 to 2000–2009. In contrast, incidence in the eastern provinces increased from a lower level of 38.8/100,000 in 1990–1999 to 65.0/100,000 in 2000–2009. Persons in this age group likely remained susceptible to measles because most were born during the period of MV introduction into the routine immunization program and scaling up of coverage [28]. These young adults born during 1970s-1990s were more likely escaped from both natural measles infection and measles vaccination, when measles incidence declined and vaccine coverage was not high during initial period of measles vaccination, especially in other province with lower development of immunization service [29]. Many your adults and migrant population did not have positive measles antibody. It was important to immunize adults susceptible to measles in Beijing. Measles vaccination campaign targeted migrant workers aged 15–40 years was conducted each year since 2005 and had a certain effect on control measles in migrant works in Beijing. Proportion of farmers, workers and farmer workers decreased from 17.8% in 2005 to 9.1% in 2010[28]. However, these populations had a strong mobility. Large-scale immunization may not interrupt measles virus transmission. For infants, the age of infection decreased slightly and the proportion of measles case increased gradually with higher vaccination coverage from 1978 in Beijing. Infants born to immune mothers received maternal antibodies transferred during the perinatal period and remained protected, on average, until approximately 4–6 months of age [30]. However, transferred maternal antibodies that were vaccine-induced rather than naturally acquired following measles infection generally existed at lower geometric mean titers in the mother and infant and waned much earlier, leaving the infant unprotected as early as 1 month of age [31,32]. In addition, the relatively high incidence among infants and young adults suggested that many young mothers may never have been vaccinated or acquired natural immunity, leaving their newborn children at increased risk from birth because they lacked protective maternal antibody against measles. The outcome of Beijing and other Western Pacific Region countries and areas will help determine whether ensuring high population immunity among adults is necessary to achieve measles elimination in other regions.

Measles incidence has varied widely by district. From 1998 to 2011, the incidence was higher in Chaoyang, Changping and Fengtai districts. Over 50% of cases were reported from these three districts. The flow of migrant workers from other provinces caused the spread of the disease. These three districts are the suburb in Beijing, in which population increase dramatically with development of economics [33]. The density of the population was higher compared with other districts [34]. Large population size and high population density negatively impact the progress of measles elimination [35]. Increasing numbers of migrant workers could be major reason for the higher incidence of measles.

To interrupt endemic transmission of measles virus, mathematical models indicate that ≥93%–95% population immunity is needed [36]. In the region of the Americas, measles was declared eliminated in 2002 after the successful implementation of a strategy that included achieving and sustaining a very high coverage (≥95%) of children [37]. Application of similar strategies in 7 southern African countries led to similar results [38]. In Beijing, the coverage of first dose MV increased steadily and reached to almost 100% from 1990. However, a gap remained between the second dose coverage and the 95% goal in 2002–2004 while SARS happened in China. The fact that the resurgence of measles incidence was found in 2005, especially for age of 1–6 years also indicated that routine coverage was insufficient to interrupt measles transmission in this age group. In addition, MV coverage rates in migrant children were only 76.6%-81.6% through investigation in 1998–2003, which significantly lower than that in local children [39-41]. SIAs among migrant children under 7 years old was implemented since 2000 in Beijing. The immunization management for migrant children after SIAs was improved greatly. However, the migrant children who lived in populous townships or had shorter residence time were still the emphasis [42-44]. Strong commitment and effort were urgently needed to maintain 95% of 2 doses routine coverage and measure coverage accurately.

This study had some limitations. Firstly, the reported MV coverage used in our analysis was likely higher than the actual MV coverage in some areas [45-47]. Secondly, it is acknowledged that due to the lack of the annual population structure in the earlier period (1957–1996), average incidence was calculated assuming the same population denominators by age group in the same stage (1957–1962, 1978, 1984, 1987-1996). Thirdly, convenience sampling was used in sero-epidemiology surveys, and the population selected may bias distribution of measles antibody level.

## Conclusions

This study showed the various effects of different strategies on the incidence of measles in Beijing, China, which would provide valuable information to commit local public health policy making and could be applied at national level. There remains a need to fully implement all components of the measles control strategy, to consider fine-tuning the strategies to sustain recent gains in measles incidence reduction and to make further progress. The emphasis is on implementing: (1) strategy to increase measles vaccine coverage in migrant adults and migrant children if transmission persists in these groups (2) routinely monitoring and validating reported vaccination coverage; (3) further research needed to better understand the changing measles epidemiology, including the role that young infants and older age groups played in sustaining measles virus transmission.

### Consent

Written informed consent was obtained from the patient’s guardian/parent/next of kin for the publication of this report and any accompanying images.

## Competing interests

The authors declare that they have no competing interests.

## Authors’ contributions

JL: (1) conception and design of the study, (2) acquisition, analysis and interpretation of data, (3) draft of the article and selection of manuscripts to discuss the results, (4) final approval of the version to be submitted; LL, XHP and MPS: (1) revising it critically for important intellectual content, (2) discussing the results, (4) final approval of the version to be submitted; RM and DLL: (1) acquisition of data, (2) analysis and interpretation of data, (3) final approval of the version to be submitted; JW: (1) revising it critically for important intellectual content, (2) editing for corrections in the English quality, (3) final approval of the version to be submitted. All authors read and approved the final manuscript.

## Pre-publication history

The pre-publication history for this paper can be accessed here:

http://www.biomedcentral.com/1471-2458/13/986/prepub
